# Geissospermiculatine, a New Alkaloid from *Geissospermum reticulatum* Bark

**DOI:** 10.3390/molecules26010143

**Published:** 2020-12-31

**Authors:** Joanna J. Sajkowska-Kozielewicz, Paweł Kozielewicz, Katerina Makarova, Marcin Stocki, Nicholas M. Barnes, Katarzyna Paradowska

**Affiliations:** 1 Department of Physical Chemistry, Faculty of Pharmacy with Laboratory Medicine Division, Medical University of Warsaw, 02-097 Warsaw, Poland; kmakarova@wum.edu.pl (K.M.); kparadowska@wum.edu.pl (K.P.); 2 Department of Physiology and Pharmacology, Karolinska Institutet, 17165 Stockholm, Sweden; pawel.kozielewicz@ki.se; 3 Institute of Clinical Sciences, College of Medical and Dental Sciences, University of Birmingham, Birmingham B152TT, UK; n.m.barnes@bham.ac.uk; 4 Institute of Forest Sciences, Faculty of Civil Engineering and Environmental Sciences, Bialystok University of Technology, 15-351 Bialystok, Poland; m.stocki@pb.edu.pl

**Keywords:** *Geissospermum reticulatum*, Apocynaceae, NMR, alkaloids, cytotoxic effects, *Danio rerio*

## Abstract

A new alkaloid, geissospermiculatine was characterized in *Geissospermum reticulatum* A. H. Gentry bark (Apocynaceae). Here, following a simplified isolation protocol, the structure of the alkaloid was elucidated through GC-MS, LC-MS/MS, 1D, and 2D NMR (COSY, ROESY, HSQC, HMBC, ^1^H-^15^N HMBC). Cytotoxic properties were evaluated in vitro on malignant THP-1 cells, and the results demonstrated that the cytotoxicity of the alkaloid (30  μg/mL) was comparable with staurosporine (10  μM). Additionally, the toxicity was tested on zebrafish (*Danio rerio*) embryos in vivo by monitoring their development (0–72 h); toxicity was not evident at 30  μg/mL.

## 1. Introduction

*Geissospermum* species (Apocynaceae) grow in the Amazonian rainforest [1,2]. For many years they have proven popular as therapeutic plants used in traditional medicines as remedies for malarial, tumors, bacterial infections, and pain relief, and as anti-inflammatory agents [1,3,4]. First investigations of the chemical composition of these trees date back to the end of the 19th century [1]. However, in recent decades, little progress has been made in the characterization of the compounds from the Geissospermum genus. In fact, among 12 species, only six (*G. argenteum, G. fuscum, G. leave, G. reticulatum, G. sericeum, and G. urceolatum*) have been studied phytochemically [1,3,5,6,7,8,9,10,11,12,13,14]. Specifically, it has been reported that these trees are a rich source of indole alkaloids [1,3]. Nevertheless, the phytochemical profile of *Geissospermum reticulatum* A. H. Gentry bark has received minimal attention with only one published study that described the presence of three alkaloids: 11-methoxygeissospermidine, flavopereirine, and geissosreticulatine [3].

As a part of our investigation into the composition and properties of plants from the Amazon region, we now document discovery of a new indole alkaloid from *G. reticulatum* bark—geissospermiculatine. Utilizing LC-MS/MS and GC-MS, the presence in the alkaloidal fraction was identified after a short isolation procedure. Moreover, the alkaloid structure was determined using 1D and 2D NMR. Finally, to assess biological properties of the compound, potential toxic effects on THP-1 cells and *Danio rerio* embryos were evaluated.

## 2. Results and Discussion

### 2.1. Structural Determination by 1D and 2D NMR Spectroscopy

Using our isolation procedure, approximately 1.8 g of the alkaloid was obtained from 100 g of *G. reticulatum* bark following. Next, we analyzed the chemical properties utilizing a range of analytical techniques. The mass spectrum of the alkaloidal fraction demonstrated that one alkaloid with a molecular ion at *m*/*z* 368 constituted 86% of the total composition (Figures S1 and S2 in Supplementary Materials). Since it was the silylated fraction, it was predicted that the molecular weight of this alkaloid was 296 g/mol. Next, the molecular formula of this alkaloid was established to be C_19_H_24_N_2_O by LC-MS/MS.

The ^1^H-NMR spectrum (in CDCl_3_) demonstrated the presence of an ethyl side chain (methyl group at *δ*_H_ 0.89 ppm, t, H-19, and methylene protons at *δ*_H_ 1.21 and 1.24 ppm, m, H-18). The ^1^H-NMR spectrum (Figure S4) also revealed four signals at *δ*_H_ 6.57 (d, *J* = 7.7 Hz, H-9), *δ*_H_ 6.74 (t, *J* = 7.4 Hz, H-10), *δ*_H_ 7.02 (t, *J* = 7.6 Hz, H-11), and *δ*_H_ 7.04 (d, *J* = 7.4 Hz, H-12) which were characteristic for aromatic moieties. The ^13^C-NMR spectrum (Figure S5), complemented by DEPT-135 and DEPT-90 experiments (Figure S6), displayed 19 signals from the alkaloid, including two methyl groups, five methylenes, eight methines, and four quaternary carbons, at *δ*_C_ 52.14 (C-7), *δ*_C_ 135.97 (C-8), *δ*_C_ 149.51 (C-13), and *δ*_C_ 172.51 (C-20).

**Figure 1:**
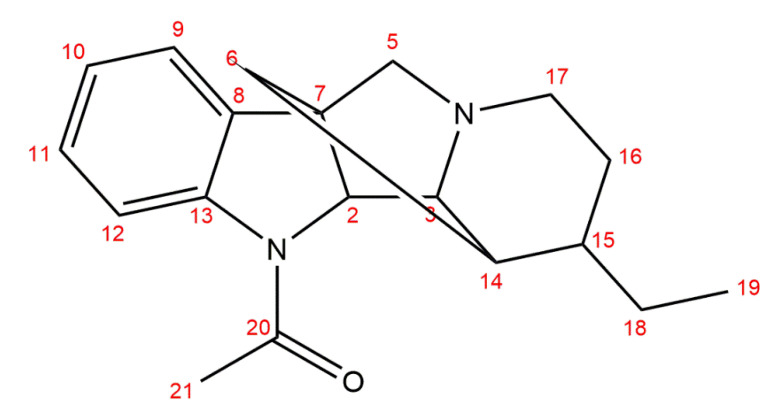
Chemical structure of geissospermiculatine.

Additionally, a range of 2D NMR spectroscopic techniques (COSY, ROESY, HSQC, HMBC, ^1^H-^15^N HMBC, Figures S7–S11) was applied to identify the structure of geissospermiculatine. The HMBC correlation networks of H-9/C-10, C-8, and H-11/C-12, C-13 indicated the linkages of C-8–C-9–C-10 and C-11–C-12–C-13, respectively. Next, HMBC analysis also showed correlations from H-2 to C-7. Moreover, the presence of a quaternary carbon at *δ*_C_ 172.51 (C-20) with one strong correlation to *δ*_H_ 2.00 (m, H-21) suggested that they formed a substituted indole ring. Correspondingly, a chemical shift of *δ*_C_ 172.51 (characteristic for R_1_CONR_2_ groups) and a chemical shift of *δ*_H_ 2.00 typical for methyl protons near a carbonyl group (CH_3_-C=O) revealed the presence of an indole ring substituted by CH_3_-C=O. In the ^1^H-^15^N HMBC spectrum, there was a correlation from H-21 (*δ*_H_ 2.00) and a nitrogen signal (*δ*_N_ 103.34), specific for primary amides, which confirmed the structure of substituent. Additionally, COSY NMR spectra displayed correlations between H-18–H-19, H-14–H-15, and H-16–H-17. The above analysis helped to determine the presence of octahydroindolizine moiety in the structure. Importantly, the presence of the bridge between C-6 and C-14 was concluded from COSY cross-peaks supported with ROESY analysis. The structure of this indole alkaloid (geissospermiculatine, Figure 1, Table 1) was, therefore, elucidated as 1-(7-ethyl-6,7,8,9-tetrahydro-6,11a-methanoindolizino[1,2-b]indol-5(5a*H*,5b*H*,11*H*)-yl)ethanone, which we named geissospermiculatine. Additionally, its three-dimensional conformation was predicted using in silico calculations.

### 2.2. Cytotoxic Activity on In Vitro Cultured Cells

**Figure 2:**
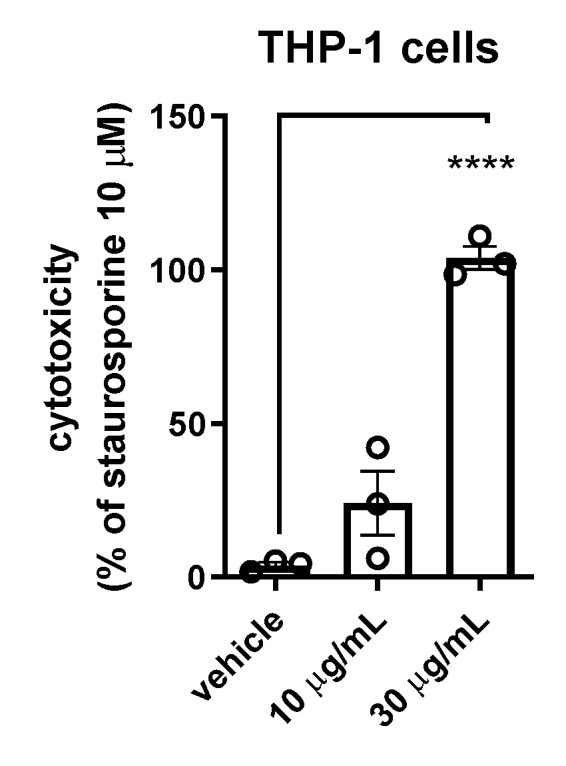
Cytotoxic properties on THP-1 cells. The data are presented as mean ± SEM from *n* = 3 independent experiments. Data were analyzed for differences by one-way analysis of variance (ANOVA). Significance level is given as **** *p*  <  0.0001.

We have previously assessed and reported cytotoxic properties of the extracts from *G. reticulatum* using THP-1 cell line [4]. This model has also been employed by others to study cytotoxic properties of plant substances, including indole alkaloids [15,16]. Our results show that the alkaloid fraction (30  μg/mL with 48 h of exposure) induced significant cell death, comparable to staurosporine (10 μM) (Figure 2). Importantly, the vehicle (0.12% ethanol) had no effect on the cell viability in this assay. Additionally, the treatment of the cells with the lower concentration (10 μg/mL) of the fraction also resulted in a decrease in the number of live cells. However, the more modest reduction did not reach statistical significance but demonstrated a concentration-dependent effect of geissospermiculatine (Figure 2). Our results suggest that the alkaloid, geissospermiculatine, could be further evaluated for the treatment of leukemia.

### 2.3. Effect on Zebrafish (Danio rerio)

The introduction of a new compound onto the market requires performing various analyses, including animal tests of toxicity. Aside from the ethical concerns raised by the public, the industry is also interested in alternative testing methods that are less time and space consuming. Fish embryos represent an attractive model for toxicological assays since they offer the possibility to perform small-scale, high-throughput analyses. Moreover, as a toxicology model, zebrafish has the potential to reveal the pathways of developmental toxicity due to their similarity with those present in mammals. Therefore, zebrafish are often considered a useful model to screen as a part of a Target Product Profile to support selection of relatively safe lead candidates early in the drug discovery process [17,18].

To test the toxicity of the fraction in vivo, we administered the alkaloid fraction to zebrafish embryos and subsequently monitored their development (0–72 h) (Table 2 and Table 3). We did not observe any evident toxic effect at 30 μg/mL—that is the concentration that caused significant THP-1 cell death in our assays (Figure 2). However, the embryos did display growth retardation upon exposure to higher concentrations of the alkaloid (100 and 300 μg/mL) (Table 3).

## 3. Materials and Methods

### 3.1. General Experimental Procedures

**Figure 3:**
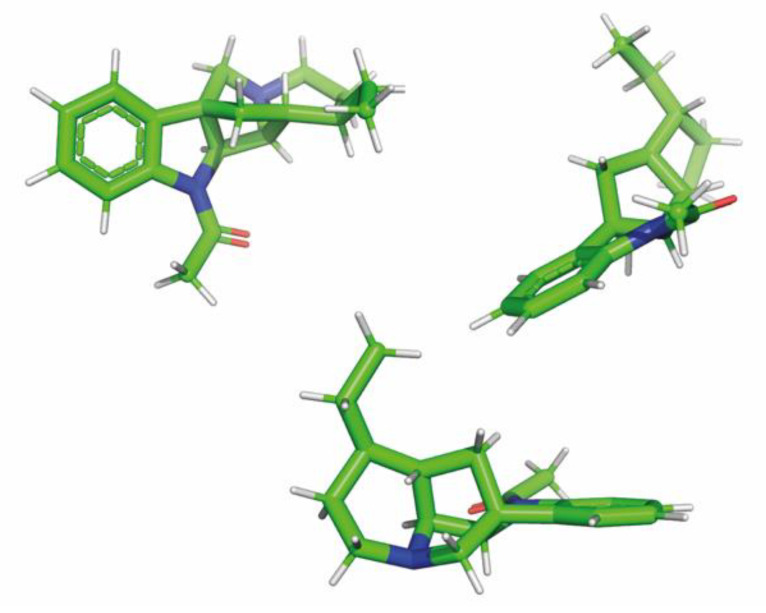
The lowest energy conformation of geissospermiculatine (optimized using HF/6-31G level of theory). Three different views of this conformation are shown.

GC-MS analysis was performed with an Agilent 7890A gas chromatograph equipped with an Agilent 5975C mass selective detector. The injection of a 1 µL sample (10 mg of extract dissolved with 1 mL of pyridine and 100 μL BSTFA was added, after which the sample was heated for 30 min at 60 °C for silylation) was performed with the aid of Agilent 7693A autosampler. The separation was performed on an HP-5MS (30 m × 0.25 mm × 0.25 µm film thickness) fused silica column at a helium flow rate of 1 mL/min. The ion source and quadrupole temperatures were 230 °C and 150 °C, respectively. The electron ionization mass spectra (EIMS) were obtained at ionization energy 70 eV. LC-MS/MS analysis was carried out by liquid chromatography (Agilent 1260 Infinity, Agilent Technologies, Santa Clara, CA, USA) coupled with a hybrid triple quadrupole/linear ion trap mass spectrometer (QTRAP 4000; AB SCIEX, Framingham, MA, USA). The 1D (^1^H, ^13^C, DEPT-135, and DEPT-90) and 2D (COSY, ROESY, HSQC, HMBC, and ^1^H-^15^N HMBC) NMR spectra were acquired on a Bruker Avance III 600-MHz spectrometer (Bruker Co., Ettlingen, Germany). The experiments were performed in a 5 mm, three-channel probe (TXI-inverse) at 295 K. Impulse sequences that came from the Bruker standard library of programs were used for the measurements. Chemical shifts for the spectra were calibrated relative to the shift of the ^1^H and ^13^C signals of the solvent (CDCl_3_). The spectra were analyzed using the MestReNova program (version 14.1.2, Mestrelab Research S.L., Santiago de Compostela, Spain) and TopSpin^TM^ (Bruker). The 3D conformation (Figure 3) was predicted of the alkaloid using the PM3 semi-empirical calculations followed by the HF/6-31G quantum mechanical calculations.

### 3.2. Plant Material

The sample of dried bark of *Geissospermum reticulatum* was collected in the Amazon rainforest and stored at National Agrarian University—La Molina (Lima, Peru) in June 2013. The identity of the plant was confirmed at the same University by Engr. Santos Jaimes Serkovic. A voucher specimen (GDMD 21760) is deposited at the Herbarium, Medical University of Gdańsk, Poland.

### 3.3. Extraction and Isolation

The process of extraction was based on that proposed by Pilarski et al. [19], with some modifications made by our team. Moreover, in comparison with Reina et al. [3], in the present study, we employed a quicker and potentially cheaper extraction protocol. Twenty mL MeOH(aq) (Chempur) was added to 1 g of minced bark and sonicated for 45 min in an ultrasonic bath (Polsonic type sonic-2) set to 45 °C. Then, 10 mL MeOH(aq) was added, and the mixture was sonicated again: for 15 min at 45 °C, and then for 45 min with switched-off ultrasound. The mixture was then filtered and evaporated to dryness. The residue was dissolved in the mixture of 10 mL of 2% H_2_SO_4_(aq) (POCH) and 10 mL of EtOAc(aq) (POCH) and sonicated for 15 min in an ultrasonic bath. The aqueous phase was separated and extracted with 10 mL of EtOAc(aq). The aqueous phase was collected and stored at 4 °C for 24 h. Then the solution was decanted from the sediment. The aqueous phase was adjusted to pH 10 with 10% NH_4_OH(aq) (Chempur), 10 mL of EtOAc(aq) was added, and the mixture was shaken (Dragon Lab shaker, Beijing, China) for 10 min. The organic phase separated, while the aqueous one was extracted again with 10 mL of EtOAc(aq). The organic fractions of alkaloids were combined and evaporated to dryness.

### 3.4. Cell Culture

THP-1 (cell line typifying human monocytic leukemia; source ATCC TIB-202^TM^) cells were cultured in RPMI-1640 medium (Sigma-Aldrich, St. Louis, MI, USA) supplemented with 1% l-glutamine (Life Technologies, Carlsbad, CA, USA), 1% penicillin/streptomycin (Sigma-Aldrich, 10,000 units penicillin and 10 mg streptomycin per mL) and 10% heat-inactivated fetal bovine serum. Cells were subcultured at a density of 1–1.5 × 10^6^ cells/mL.

### 3.5. Cytotoxic Activity Test with 7-AAD

Cells were stimulated for 24 h and, following the incubation, they were washed with PBS by centrifugation at 400× *g* for 5 min. The supernatant was discarded, and the cells were resuspended in 7-AAD solution (50 μg/mL, eBioscience, San Diego, CA, USA) and incubated for 20 min at 4 °C. Cells were kept on ice and immediately analyzed on an ADP Cyan flow cytometer (Beckman Coulter, Brea, CA, USA). The flow cytometry data were analyzed using FlowJo V10 (Tree Star, Ashland, OR, USA).

### 3.6. Zebrafish (Danio rerio) Embryo Toxicity Test

The zebrafish embryo toxicity test was performed according to published guidelines. It should be noted that according to the EU Directive 2010/63/EU on the protection of animals used for scientific purposes, the earliest life stages of animals are not defined as protected and, therefore, do not fall into the regulatory frameworks dealing with animal experimentation. Zebrafish (*Danio rerio*) used in this experiment were of the wild-type AbxTL strain. The fish were housed in a circulating system that continuously aerates and filters the water to maintain it in the right quality. The temperature in the room and tanks was maintained between 26.0–28.5 °C (pH 6.8–7.5) on a 14 h light:10 h dark cycle. For all experiments, E3 medium (5 mM NaCl, 0.17 mM KCl, 0.33 mM CaCl_2,_ and 0.33 mM MgSO_4_) was used. Twenty fertilized eggs of 4 h postfertilization (hpf) were used for each experiment. The selected eggs were exposed to 5 mL of alkaloid fraction of 100 µg/mL and 300 µg/mL *Geissospermum reticulatum* dissolved in E3 or E3 alone as a control.

All experiments were performed in duplicates. E3 controls were performed with each set of experiments. The samples were incubated at 28 °C for 24, 48, and 72 h and embryonic development was observed with a Leica M165 microscope after exposure to the studied environment. The observations were made at room temperature (20 °C). The delay in the development, the number of spontaneous movements (in 20 s), heartbeats, length of each fish, development of ears, size of the pericardium, tail deformation, and mortality were evaluated. The delay in the development was estimated based on the percent of embryos not developed to the same stage as those treated with E3. To measure the heart rate, individual embryos were placed under the microscope, and then a 10-s lapse video was recorded. The number of heartbeats was recalculated to the beats per minute. To measure the length of fish or the size of the pericardium, a photo was taken while individual embryos were placed under the microscope. Other observations were appraised visually. The figures were analyzed using the ImageJ program.

## 4. Conclusions

In comparison with the previous report, here, we employed an optimized (faster and potentially more cost efficient) protocol that enabled us to extract a novel indole alkaloid (geissospermiculatine). The compound exhibited cytotoxic properties upon malignant THP-1 cells in vitro, yet did not cause substantial defects of normal zebrafish embryos at a THP-1 cell cytotoxic concentration.

## Figures and Tables

**Table 1:** NMR data and HMBC correlations for geissospermiculatine (*δ* in ppm) ^a^.

	Compound		
Position	*δ*_H_ (mult., *J* in HZ)	*δ*_C_	HMBC ^b^
2	3.97 (d, 5.5)	64.44	6, 7, 14
3	1.91 (br s)	33.76	
5	2.99 (m) and 3.47 (m)	54.75	7, 16
6	2.17 (ddd, 11.6, 11.1, 3.4) and 2.37 (m)	37.93	2, 5, 7
7		52.14	
8		135.97	
9	6.57 (d, 7.7)	109.75	8, 10
10	6.74 (d, 7.4)	119.47	8, 9
11	7.02 (d, 7.6)	128.01	12, 13
12	7.04 (d, 7.4)	122.30	11, 13
13		149.51	
14	3.38 (m)	66.35	
15	1.66 (dt, 9.9, 14.7)	41.38	
16	2.40 (m) and 3.04 (s)	49.28	14, 15
17	1.69 (dt, 9.9, 14.7) and 2.21 (m)	25.27	
18	1.21 and 1.24 (m)	23.70	15, 16
19	0.89 (t)	11.45	15, 18
20		172.51	
21	2.00 (m)	22.59	20

^a^ Measured at 600 (^1^H) and 150 (^13^C) MHz in CDCl_3_; ^b^ HMBC correlations are from proton(s) stated to the indicated carbon.

**Table 2:** Zebrafish embryo mortality.

Substance	E3 [%]	Alkaloidal Fraction	
Time		100 µg/mL [%]	300 µg/mL [%]
24 h	2	2.5	50
48 h	0	0	0
72 h	0	0	100

**Table 3:** Toxicological effects during 72 h embryonic development of *Danio rerio.*

	Substance	E3 [%]	Alkaloidal Fraction	
	Development Features		100 µg/mL [%]	300 µg/mL [%]
24 h		*n* = 8	*n* = 39	*n* = 20
	length [%]	not hatched	not hatched	not hatched
	heartbeats [/min]	132 ± 3	139 ± 2 *	125 ± 2
	pericardial edema [%]	none	none	none
48 h		*n* = 8	*n* = 7, 32 not hatched	*n* = 6, 14 not hatched
	length [%]	reference (100%)	↓ 5% *	↓ 12% *
	heartbeats [/min]	154 ± 5	133 ± 3 *	44 ± 2 *
	pericardial edema [%]	reference (100%)	↑ 8%	↑ 46%
72 h		*n* = 8	*n* = 8, 1 not hatched	*n* = 8
	length [%]	reference (100%)	↓ 16% *	death
	heartbeats [/min]	not measured	not measured	death
	pericardial edema [%]	reference (100%)	↑ 33%	death

* Significantly different values; h, hours; ↓ decrease compared with E3; ↑ increase compared with E3.

## Data Availability

The data presented in this study are available on request from the corresponding author.

## Supplementary Materials

The following are available online. Figure S1: GC-MS chromatogram of studied fraction after the silylation process. Figure S2: Mass spectrum of silylated geissospermiculatine (Rt = 58.124 min.) from GC-MS analysis. Figure S3: Significant HMBC (in black) and COSY (in blue) correlations. Figure S4: ^1^H-NMR spectrum of geissospermiculatine. Figure S5: ^13^C-NMR spectrum of geissospermiculatine. Figure S6: DEPT-135 (A) and DEPT-90 (B) NMR spectra of geissospermiculatine. Figure S7: COSY spectrum of geissospermiculatine. Figure S8: ROESY spectrum of geissospermiculatine. Figure S9: HSQC spectrum of geissospermiculatine. Figure S10: HMBC spectrum of geissospermiculatine. Figure S11: ^1^H-^15^N HMBC spectrum of geissospermiculatine.

## Sample Availability

Bark samples are available from the authors.
