# Supplementary material for: Geissospermiculatine, a New Alkaloid from Geissospermum reticulatum Bark
Source: Molecules. 2020 Dec 31;26(1):143. doi: 10.3390/molecules26010143 (PMC7795935; doi:10.3390/molecules26010143)
Supplement: Supplementary file 1 [file molecules-26-00143-s001.pdf]

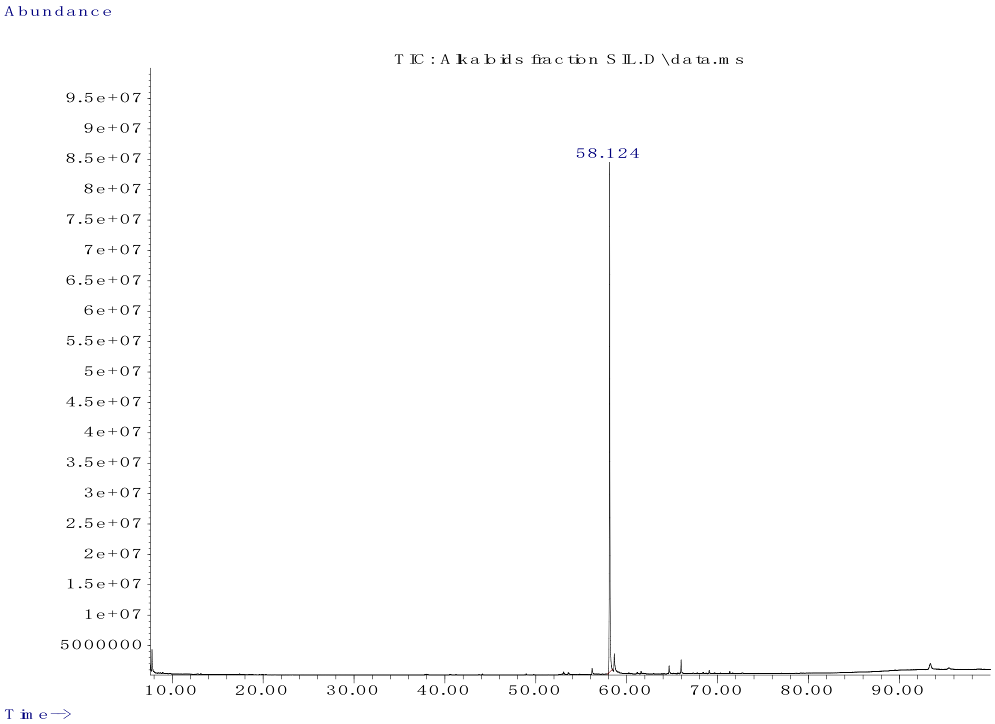

Figure S1. GC-MS chromatogram of studied fraction after the silylation process.

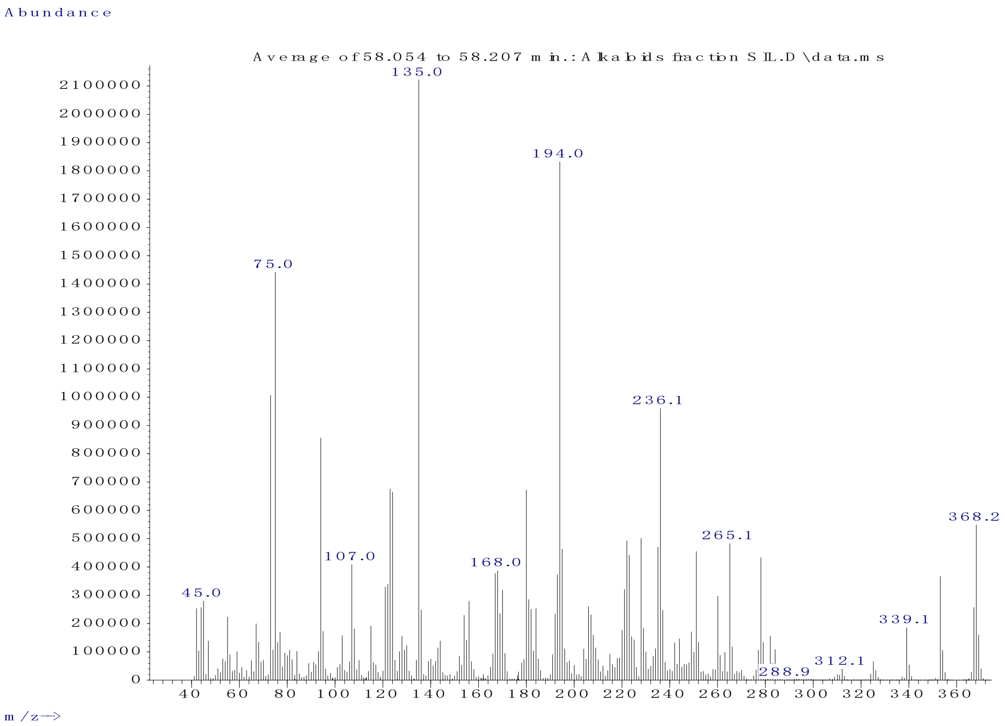

Figure S2. Mass spectrum of silylated geissospermiculatine (Rt = 58.124 min.) from GC-MS analysis.

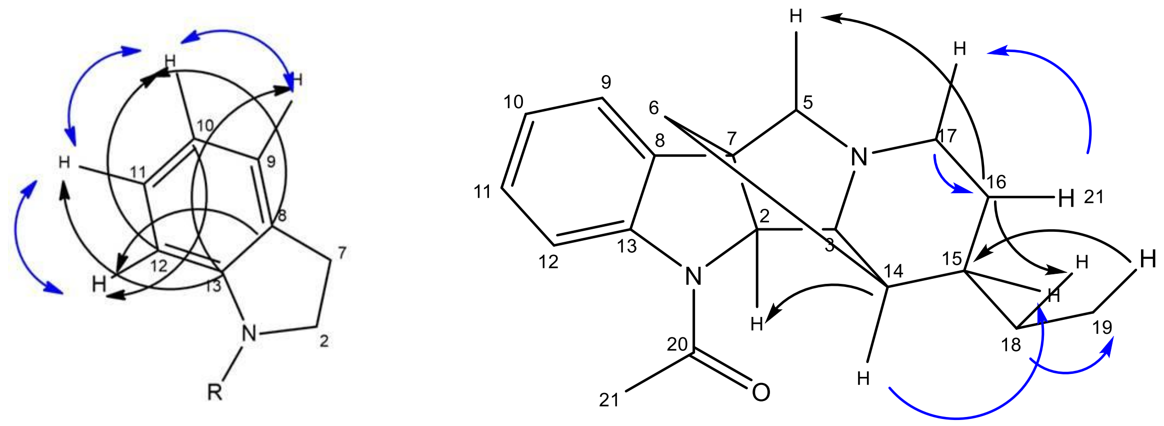

Figure S3. Important HMBC (in black) and COSY (in blue) correlations of 4.

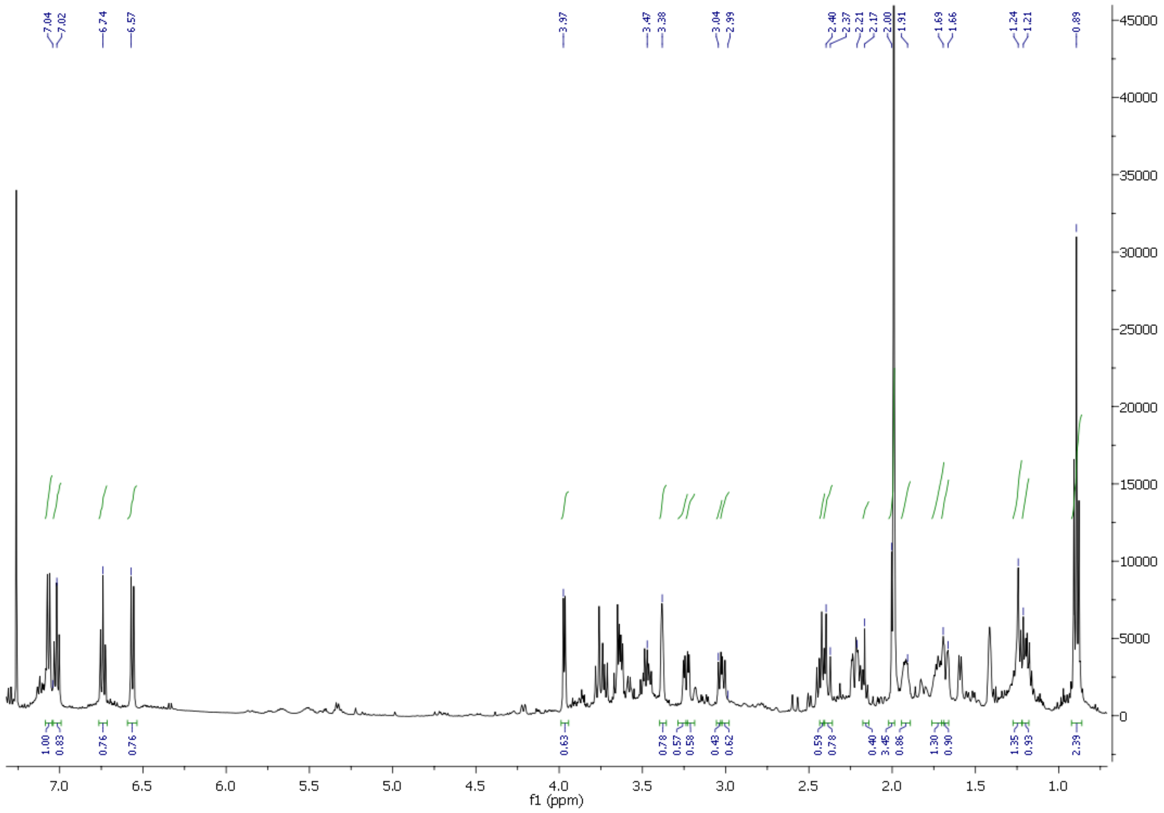

Figure S4. ^{1}H NMR spectrum of geissospermiculatine.

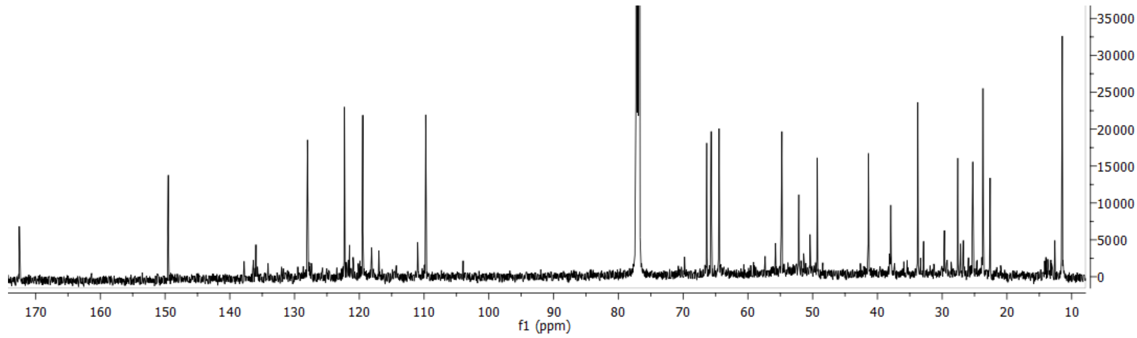

Figure S5. ^{13}C NMR spectrum of geissospermiculatine.

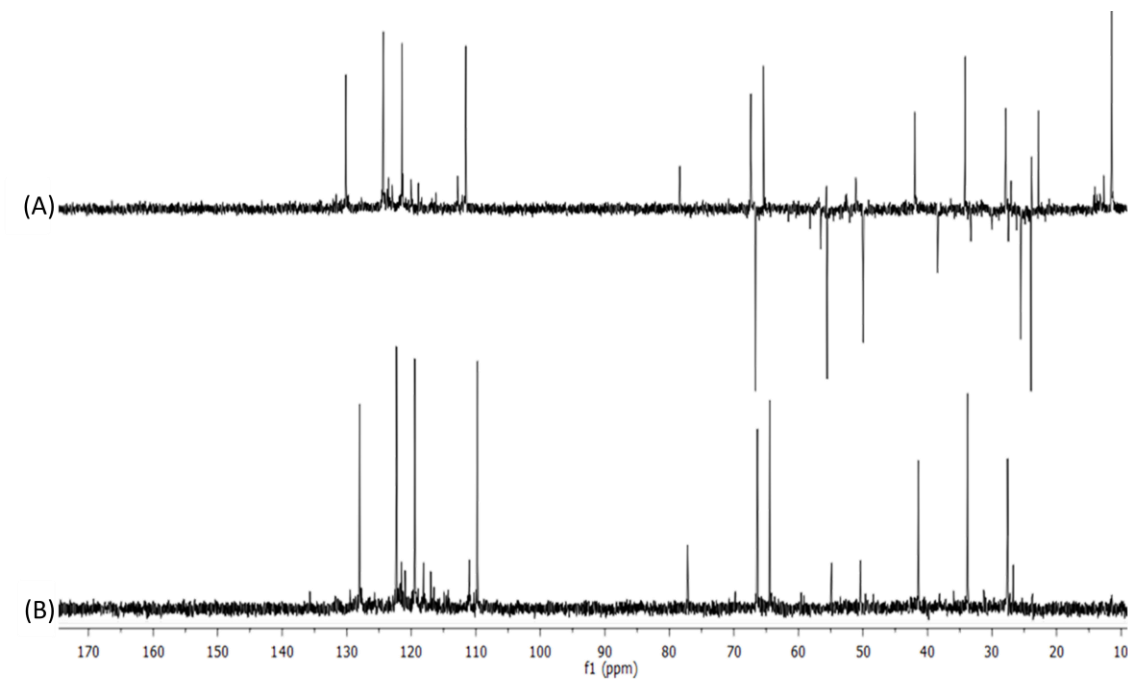

Figure S6. DEPT-135 (A) and DEPT-90 (B) NMR spectra of geissospermiculatine.

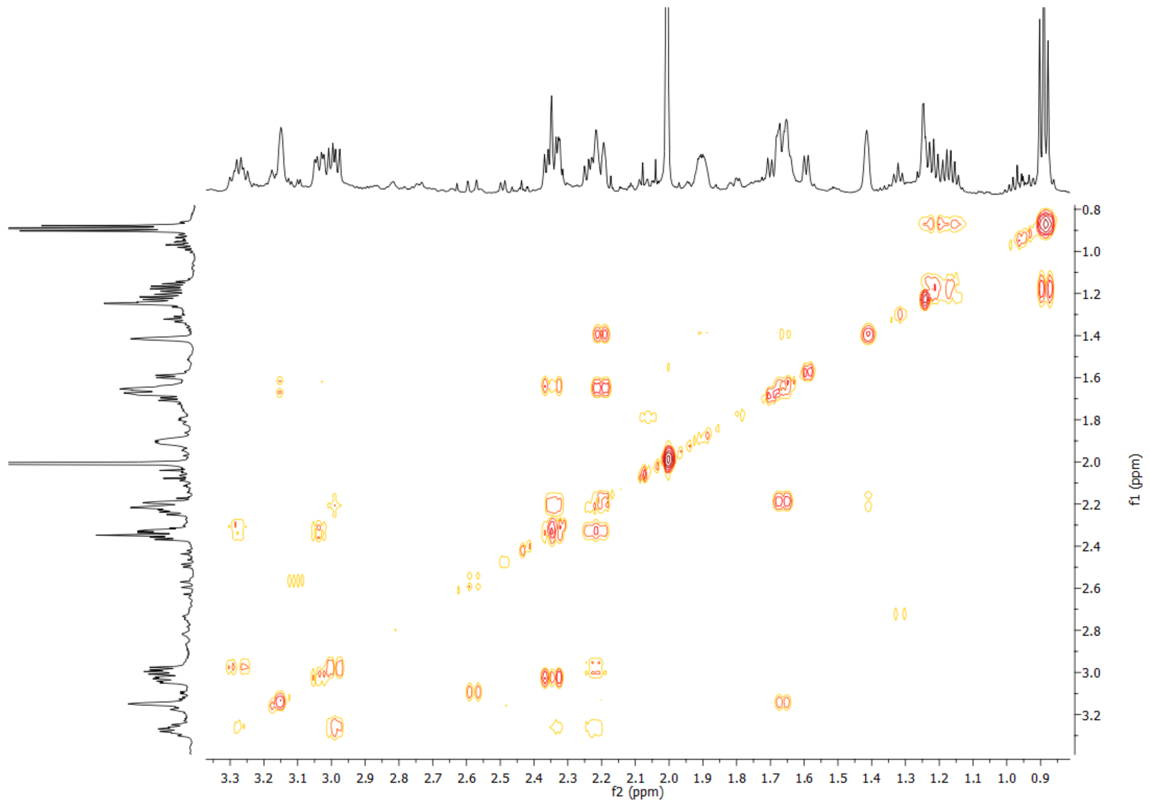

Figure S7. COSY spectrum of geissospermiculatine.

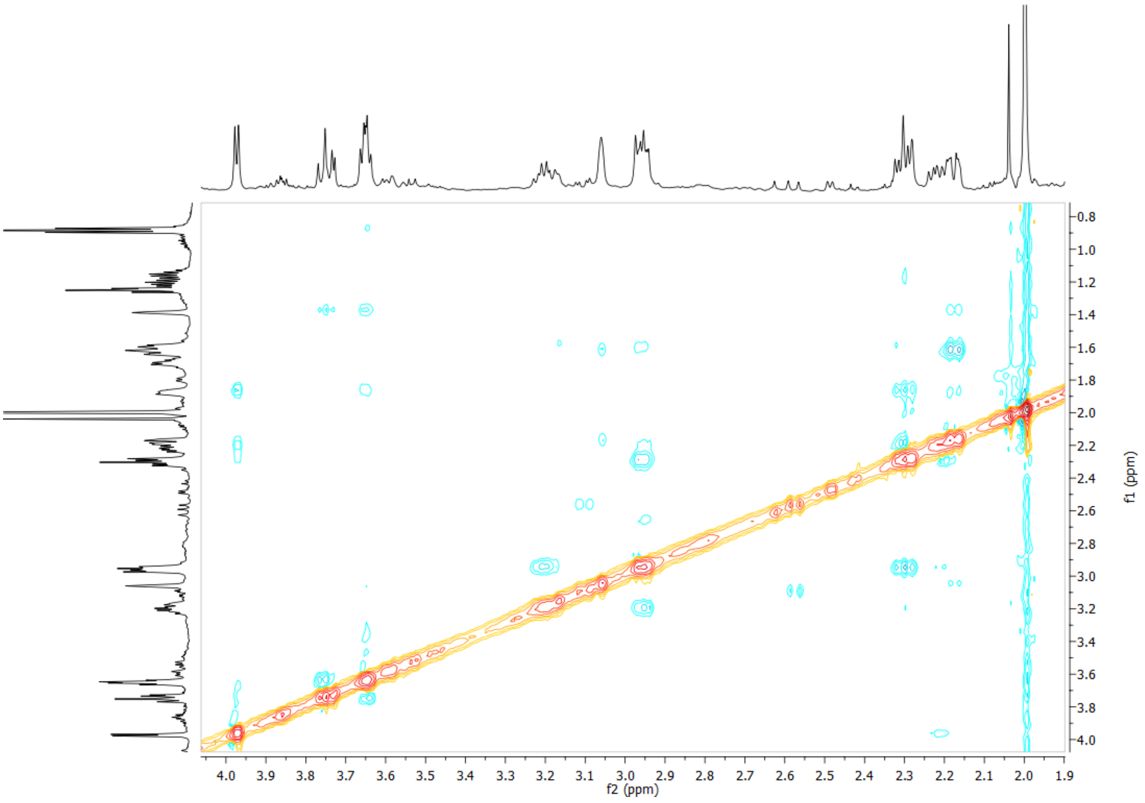

Figure S8. ROESY spectrum of geissospermiculatine.

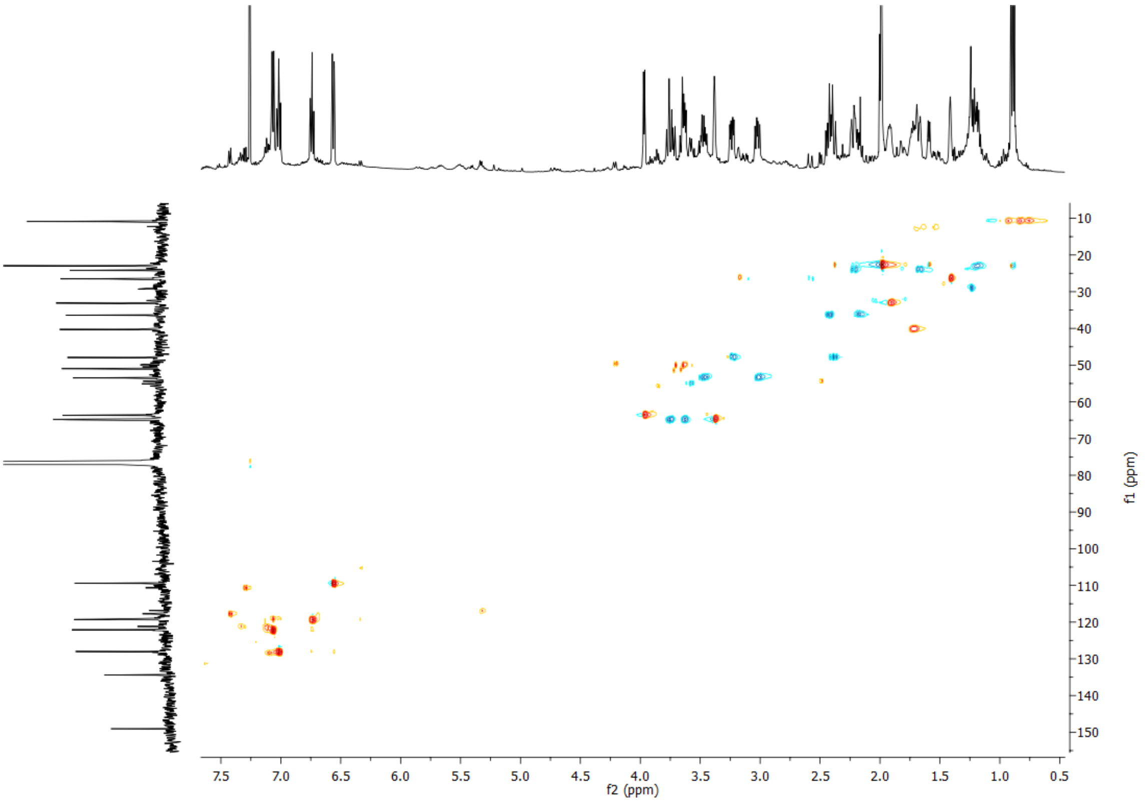

Figure S9. HSQC spectrum of geissospermiculatine.

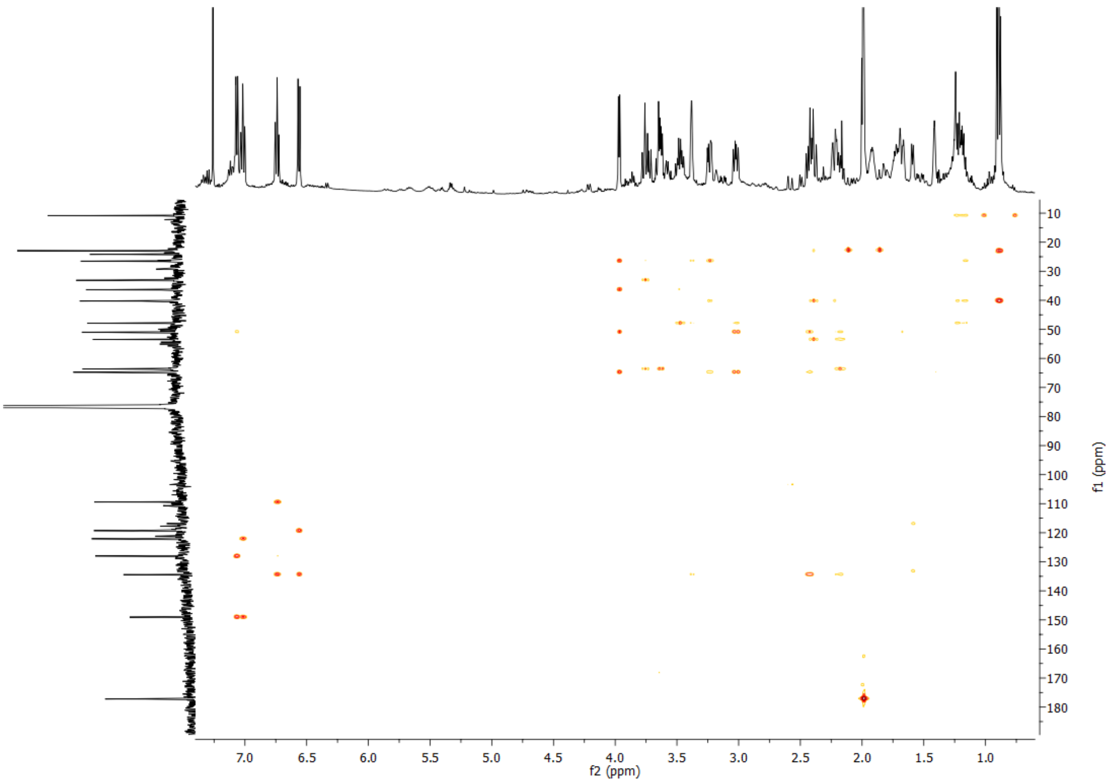

Figure S10. HMBC spectrum of geissospermiculatine.

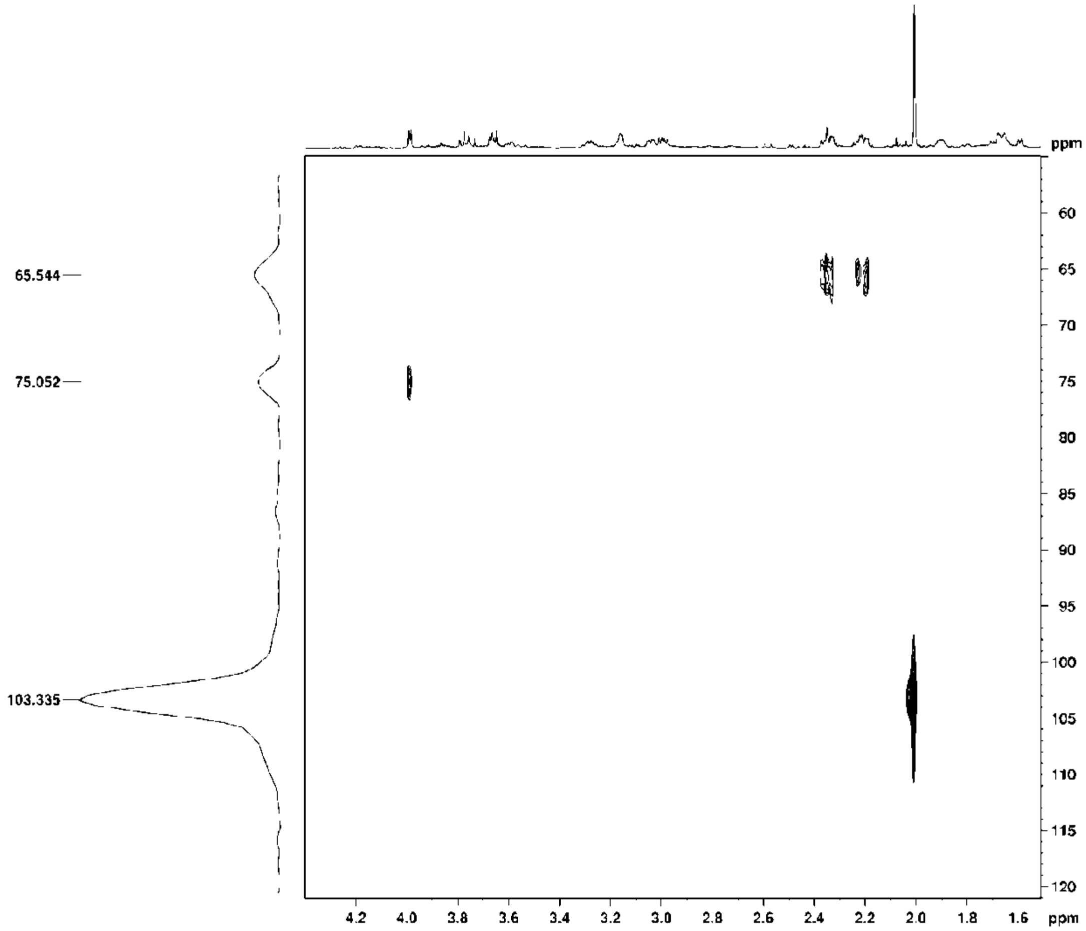

Figure S11. ^{1}H-^{15}N HMBC spectrum of geissospermiculatine.
